# Soft palate preservation after tumor resection with transoral laser microsurgery

**DOI:** 10.4317/medoral.18634

**Published:** 2012-12-10

**Authors:** Kuauhyama Luna-Ortiz, Antonio Gómez-Pedraza, Adalberto Mosqueda-Taylor

**Affiliations:** 1 MD, MD. Departamento de Cirugía de Cabeza y Cuello, Instituto Nacional de Cancerología, Mexico, D.F., México; 2 DDS, MSc. Departamento de Atención a la Salud, Universidad Autónoma Metropolitana Xochimilco, México, D.F., México; 3 ….

## Abstract

Background: Management and preservation of the soft palate is dependent on clinical stage and tumor histology. However, available literature is scarce regarding the palate preservation with the use of laser CO2. Objectives: We report the results obtained after management with laser surgery and soft palate preservation in three patients with salivary gland neoplasms. Method: Three patients with minor salivary gland tumors were treated by means of transoral laser microsurgery. All tumors were assessed using magnetic resonance imaging. All tumors were >3 cm. Soft palate function was preserved and reconstruction was performed with primary closure. Patients began oral feeding the same day and were discharged after 24 h. Conclusions: Transoral laser microsurgery is recommended for treatment of soft palate tumors. This treatment can be considered a better option when compared with other modalities such as radio- or chemoradiotherapy which require a longer time of treatment, are more expensive and tend to produce significant toxicity.

** Key words:**Laser CO2, neoplasms, salivary gland.

## Introduction

Primary tumors of the soft palate are rare. They present up to 10 times less often than laryngeal carcinomas and up to five times less often than tonsillar neoplasias. (1,2) Management and possibility of conservation of the soft palate depends on clinical stage and tumor histology. (3) In this regard, there are several case reports in which reconstruction is carried out with free flaps for bulky tumors that extensively involve this region. In contrast, the attempt at conservation by marginal surgery with the use of new technologies such as transoral laser microsurgery and/or robotic surgery has not been properly explored. (4) The aim of this study is to report the results obtained after management with laser surgery and soft palate preservation in three patients with salivary gland neoplasms.

Patient and Methods

We present a series of three cases of minor salivary gland tumors located in the mucosa of the soft palate. In all cases, the diameter was assessed by magnetic resonance imaging studies (MRI), with all tumors being >3 cm ( Table 1). All patients underwent transoral microsurgery with CO2 laser under general anesthesia with oral intubation. It was possible in all cases to preserve soft palate function and reconstruction was performed with primary closure. Patients began oral feeding on the same day and their hospital stay was only 24 h.

**Table 1:**
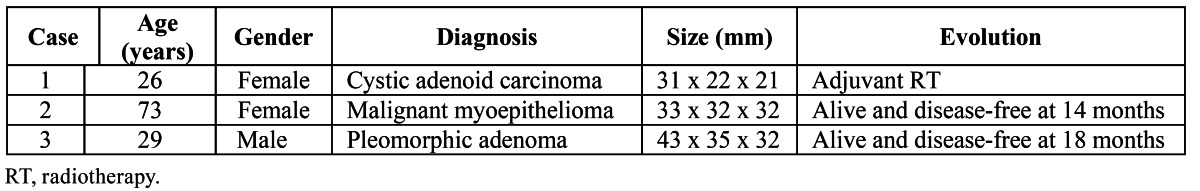
Clinicopathological characteristics of patients treated with transoral laser CO2 microsurgery.

Case 1

**Figure 1:**
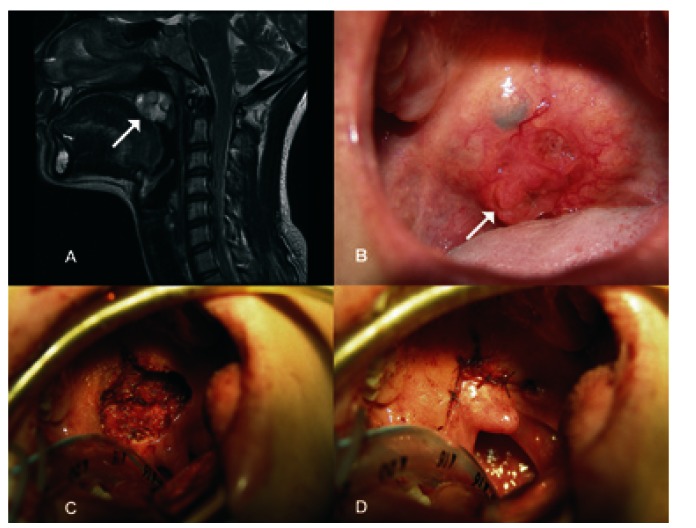
A) Sagittal magnetic resonance imaging (MRI) showing dependent lesions of the soft palate shaping the dome of the tongue at the base. B) Photograph of the lesion showing ulceration. C) Surgical bed after resection with CO2 laser. D) Primary closure of the palate defect.

A 26-year-old female was referred to our institution after a biopsy of the soft palate with a diagnosis of adenoid cystic carcinoma. The patient’s condition began 6 years prior with the appearance of a soft palate lesion. For 2 years the patient experienced bleeding and odynophagia, which prompted the present consultation. Upon initial physical examination a nasofibrolaryngoscopy was performed. No data of involvement of the nasal portion of the soft palate was noted, but an exophytic lesion occupying the entire length of the soft palate without clinical extension to the tonsillar pillars or hard palate was found. The neck was clinically negative. Magnetic resonance imaging (MRI) was performed and showed the presence of a tumoral growth that measured 31 x 22 x 21 mm and partially obliterated (~30%) the width of the oronasopharynx contiguously with the dome of the tongue (Fig. 1). We performed excision of the lesion with CO2 laser with grossly negative margins and primary closure (Fig. 1). Macroscopical examination of the tumor showed a lesion measuring 2.5 x 2 x 1.8 cm. Histopathological report was adenoid cystic carcinoma G2 with surgical borders free of tumor and presence of thermal damage. One month after the procedure, the patient has adequate function of the palate and barely perceptible nasal voice. She was treated with adjuvant radiotherapy due to histology and tumor grade. After one year she is disease free.

Case 2

**Figure 2:**
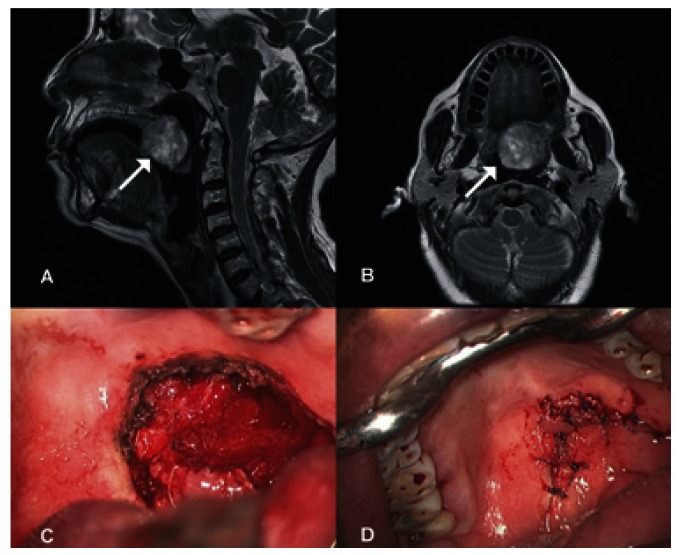
A) Sagittal MRI showing lesion in the soft palate without compromise of the nasal cavity. B) Axial MRI with lesion found to the left of the midline, dependent on the soft palate. C) Photo after resection showing the defect in the soft palate. D) Photograph showing primary closure in the immediate postoperative period.

A 73-year-old female was referred due to a 3 year-duration soft palate lesion. The patient began experiencing pain 15 days prior to her first consultation at our institution. On admission, physical examination disclosed a soft palate tumor measuring 4 x 3 cm. The lesion was ulcerated in the center with raised borders and areas of erythema, which extended up to the left tonsillar pillar. On nasofibrolaryngoscopy it was observad that the lesion was displacing the soft palate. MRI was performed, demonstrating an expansive tumoral lesion located in the posterior and inferior border of the soft palate with well-defined edges of ~33 x 30 x 30 mm. The lesion completely obliterated the lumen of the naso-oropharynx (Fig. 2). Surgery was performed with CO2 laser for preservation and primary closure of the soft palate (Fig. 2). Final histopathology report was minimally invasive malignant myoepithelioma (<1.5 mm), which probably originated as pleomorphic adenoma with lymphatic and vascular permeation and necrosis. Tumor size was 3.8 x 3 x 2.8 cm and surgical margins were free of neoplasm. At 14-months of follow-up, the patient demonstrates no clinical evidence of tumor activity and no functional impairment.

Case 3

**Figure 3:**
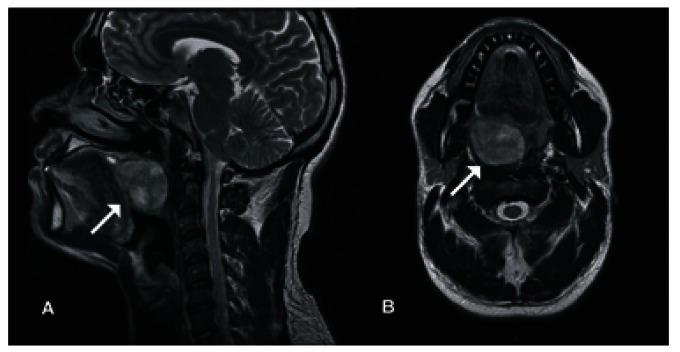
A) Sagittal MRI showing lesion in the soft palate and there is total obstruction of the naso-oropharynx. B) Axial MRI with lesion found to the right of the midline dependent on the soft palate.

A 29-year-old male with no relevant medical history was referred due to the presence of a tumor located in the soft palate which produced sensation of a foreign body and has been present for 1 year. Physical examination showed an ulcerated 3-cm diameter tumor. MRI revealed a predominantly solid lesion with some linear punctate hyperintense areas located in the soft palate and anterior pillar of the oropharynx in the right parasagittal margin, which measured 40 x 39 mm and caused a decrease in the amplitude of the oropharynx (Fig. 3). Under general anesthesia, surgical excision of the lesion with transoral laser surgery and primary closure for preservation of the soft palate was performed. The pathology report informed a diagnosis of pleomorphic adenoma. The surgical specimen measured 4.3 x 3.5 x 3.2 cm with neoplasm in close proximity to the surgical margins, and for this reason surveillance of the patient was decided upon. At 18 months postoperatively, the patient demonstrated no evidence of tumor activity, and function of the soft palate is preserved.

## Discussion

The soft palate is one of the subsites of the oropharynx with a greater degree of specialization. Its physiological importance lies in the movement that it is able to perform. It also serves as an anatomic barrier, which allows the individual to perform functions such as swallowing, speech and breathing because it prevents the passage of air or food into the nasal cavity. This dynamic anatomic physiology is altered when tumors involve this important structure of the oropharynx, such as carcinomas originating from the oral and sinus mucosa and tumors of minor salivary glands that may develop as occupying masses altering the normal activity of this structure.

Primary tumors of the soft palate and uvula are rare compared to other oral tumors. Their treatment is commonly considered in conjunction with tumors in the oropharynx, without a distinction in the subsite where they are located. (5-8) Surgery for these types of lesions poses a major challenge due to the marked dysfunction that may result from ablation of the soft palate, with consequences such as rhinolalia and nasal reflux. This contrast with the effectiveness of radiotherapy as initial treatment in some early-stage malignancies and the addition of chemotherapy in more advanced-stage malignancies.

In recent years the availability of new technologies has modified this approach as reported recently by Grant et al. (9) who used laser resection in the treatment of tumors of the oropharynx with encouraging results. Adjuvant therapy according to pathological findings is not modified. Instead, improved staging of the primary tumor with surgical exposure is achieved and, consequently, provides a better clinical evaluation, resulting in shorter treatment duration with less morbidity and excellent functional outcomes compared with chemo- or radiation therapy-based management.

Although transoral microsurgery has not been frequently used for these types of lesions, studies dating back to the 1990´s report that its use was already implemented. It is unclear whether such cases were aimed at soft palate and/or uvula preservation as a measure for conservation of function and, consequently, a better quality life. (10) In the study by Parsons et al. (5) the authors concluded that surgery (with or without adjuvant radiotherapy) has a higher morbidity than radiotherapy alone compared as primary treatment, which has now been challenged by the low morbidity produced when using laser resection. (6) Thomas et al. (11) demonstrated that there is voice dysfunction in >70% of patients with tumors of the oropharynx, which appears to be related to radiotherapy treatment, the presence of free flaps and tumor stage. Surgical treatment has routinely been designed to include wide margins, which usually involves ablation of the soft palate. Therefore, the most optimal method for reconstruction in recent decades has been anatomic placement of a barrier such as microvascular free flaps often from the anterolateral thigh region. (12) This reduces the problem only from the anatomic point of view as a barrier, but rehabilitative function related to movement of the soft palate has not been previously possible, continuing with one or more of the prominent consequences related to lack of the soft palate.

According to the results found in this study, there are several reasons for proposing treatment with transoral microsurgery as a better option over conventional therapies. First, all neoplasms in our cases were of minor salivary gland origin, in which surgery remains as the best management. Second, the short duration of the procedure is performed without affecting swallowing and allows discharge of the patient in the first 24 hours, which does not occur with radiation therapy, as this treatment is longer and do not necessarily produce a favorable response in tumor size and usually produce higher economical costs for the patient. Although controversy exists as to the assessment of margins in laser microsurgery, we agree that the principle of this treatment is precisely to provide better intraoperative clinical assessment where the margins are decided upon by the surgeon using the microscope, which translates into greater security for providing an adequate surgical margin.

## Conclusions

Transoral laser microsurgery and, in the near future robotic surgery, will play a greater role in the treatment of soft palate tumors and, in general, for tumors of the oropharynx due to the adequate exposure achieved with these methods. This allows improved clinical assessment of lesions, especially in these locations that, until recently, were difficult to access with conventional open surgery or required approaches that resulted in high morbidity. Based on the aforementioned, this treatment should be considered a better choice as compared with techniques such as radio- or chemoradiotherapy that require a longer time of treatment, are more expensive and tend to produce significant toxicity.
